# Felbamate as a therapeutic alternative to drug-resistant genetic generalized epilepsy: a systematic review and meta-analysis

**DOI:** 10.1007/s10072-024-07942-6

**Published:** 2024-12-26

**Authors:** Yitao Ma, Matthew Kaminski, Robert Crutcher

**Affiliations:** 1 Department of Neurology, Walter Reed National Military Medical Center, Bethesda, MD 20889 USA; 2 University of Maryland, College Park, MD 20742 USA; 3 The Division of Neurology at Nemours, Mayo Clinic, Jacksonville, FL 32207 USA

**Keywords:** Felbamate, Drug-resistant epilepsy, Genetic generalized epilepsy, Idiopathic generalized epilepsy

## Abstract

**Introduction:**

The effect of felbamate (FBM) on genetic generalized epilepsy (GGE) remains largely unknown. The utilization of FBM has been limited due to its potential risk of aplastic anemia and hepatic failure. This study aimed to comprehensively evaluate the efficacy and safety of FBM in the treatment of drug-resistant GGE.

**Methods:**

We searched the databases, including PubMed, Web of Science, Embase, and Google Scholar, to identify cases of GGE treated with FBM. Data on outcomes and adverse events were extracted from these studies.

**Results:**

The literature search yielded 9 studies with 166 cases in which FBM was used as an adjunct therapy to treat drug-resistant GGE. The pooled responder rate to FBM was 65% (95% confidence interval CI, 51–80). 17% (95% CI, 3–31) achieved seizure freedom. 81% (95% CI, 60–100) of patients with Epilepsy with myoclonic atonic seizures were responders. Adverse events were reported in 40% (95% CI, 26–54) of patients.

**Conclusions:**

Patients with drug-resistant GGE achieved good responses to FBM. The high heterogeneity between studies calls for further research with large-scale, randomized controlled trials. Given the rare reports of idiosyncratic reactions of aplastic anemia and hepatic failure, intense laboratory monitoring and a slower titration schedule are recommended.

**Supplementary Information:**

The online version contains supplementary material available at 10.1007/s10072-024-07942-6.

## Introduction

In 2017, the International League Against Epilepsy (ILAE) classified “genetic generalized epilepsies” (GGEs) as a broad group of epilepsies with generalized seizure types and generalized spike-wave discharges on electroencephalography (EEG) [1]. These epilepsies are thought to have a genetic etiology based on findings from twin and family studies [2]. GGEs make up around one-fourth of all epilepsies [3].

Idiopathic generalized epilepsies (IGEs) are a subgroup of GGEs. IGEs comprised of four syndromes: childhood absence epilepsy (CAE), juvenile absence epilepsy (JAE), juvenile myoclonic epilepsy (JME), and epilepsy with generalized tonic-clonic seizures alone (GTCA) [4]. Some patients may shift from one phenotype to another. These are the most prevalent syndromes among the GGEs, impacting around 15–20% of individuals with epilepsy [5]. The majority of patients with IGE have a good prognosis, with remission rates ranging from 64 to 82% [6]. However, a recent study conducted by Jiang’s group revealed that the pooled prevalence of drug-resistant epilepsies (DRE) in IGE cohorts was 27% [7]. While studies from Gomez-Ibanez and Cação groups indicated that up to 50% of IGE cases exhibited resistance to standard anticonvulsant therapy [8, 9].

In addition to IGE, GGEs also include individuals with generalized seizure types who do not meet the criteria for a specific syndrome and less common generalized epilepsy syndromes. They can be associated with a developmental and epileptic encephalopathy (DEE) or an epileptic encephalopathy (EE). These include epilepsy with myoclonic-atonic seizures (EMAtS), epilepsy with myoclonic absences (EMA), epilepsy with eyelid myoclonia (EEM) and myoclonic epilepsy in infancy (MEI), etc. EMAtS is a primary generalized idiopathic epilepsy syndrome characterized by prominent myoclonic and astatic seizures. EMAtS has a historical reputation for being difficult to treat. Multiple anti-seizure medications (ASMs), the ketogenic diet, and other therapies including surgery have been attempted for the management of EMAtS. EMA is another childhood generalized epilepsy syndrome with a high rate of drug resistance [10, 11]. 45% of patients also experience GTCs [12]. 70% of patients may experience cognitive deterioration and evolve into other forms of epilepsy.

Felbamate (2-phenyl-1,3-propanediol dicarbamate) was initially produced in 1954 by Wallace Laboratories [13]. It was approved by the United States Food and Drug Administration (FDA) in 1993 to treat adults with focal seizures with or without secondary generalization and children with Lennox–Gastaut syndrome (LGS) [13]. Extensive clinical trials and immediate post-marketing experience proved that felbamate (FBM) is an anti-convulsant with high efficacy. FBM was found to be effective in treating epilepsy that had been resistant to other ASMs [13]. Although preliminary studies have demonstrated that felbamate may be beneficial for generalized epilepsy, no controlled studies are available [14, 15]. FBM has been associated with multiple reported cases of aplastic anemia (AA) and acute hepatic failure and its use is now restricted. A joint practice advisory committee from the American Academy of Neurology and the American Epilepsy Society recommended considering FBM solely for individuals with seizures resistant to other ASMs [16]. Subsequent evaluations from multiple centers have questioned the need for such restrictions [17–20]. Because of its strong therapeutic index, many epileptologists continued to prescribe FBM for patients with severe epilepsy. This study aims to summarize the current literature concerning the efficacy and safety of FBM for treating drug-resistant GGE.

## Methods

This systematic review was reported following the guidelines of the Preferred Reporting Items for Systematic Reviews and Meta-Analyses (PRISMA) statement [21].

Relevant studies were retrieved from Pubmed, Web of Science, Embase, and Google Scholar indexed from inception to April 2024. Two reviewers (YM and MK) independently carried out the search process for each of the mentioned databases. Individual queries for each database were performed by systematically applying and replicating the search terms across all sources. The search terms included “felbamate” or “felbatol” and “epilepsy” or " generalized epilepsy” or “myoclonus” or “absence seizures”. Data of patients with GGE treated with FBM were extracted from the included studies. All variables, including study design, age, gender, dosage, seizure types, responder rate, and adverse events (AEs), were documented in a Microsoft Excel spreadsheet. Responders are defined as > 50% seizure reduction or seizure freedom. All reported AEs were collected to summarize the most common AEs.

The inclusion criteria were original research papers that studied the outcomes of patients with GGE treated with FBM. Only articles in English were reviewed and included.

The exclusion criteria included reviews, editorials, commentaries, animal studies, case reports, conference proceedings, studies of other epilepsy types than GGE, studies of combined generalized and focal epilepsy such as Dravet syndrome or Lennox Gastaut syndrome, studies not related to the treatment of epilepsy or having no relevant or limited presentation of findings. Titles, abstracts, and full-text articles were thoroughly screened by two reviewers for compliance with the inclusion criteria. Any discrepancies or disagreements were resolved through further discussion. The risk of bias was evaluated by the Newcastle-Ottawa Quality Rating Scale (NOS) [22]. The level of evidence was graded using the Grading of Recommendations Assessment, Development, and Evaluation (GRADE) working group criteria [23].

Statistical analysis was performed using R (version 4.2.2) and the *meta* and *metafor* packages. The overall pooled estimate of the outcomes and AE rates was computed using the “metaprop” command and the random-effects model for meta-analysis. Additional subgroup analyses were performed according to different epilepsy types. Forest plots were used to display the estimated variance between studies, their 95% confidence interval (CI), and weights for each study. The statistical heterogeneity was measured using the *I*^*2*^ statistic and values of 25%, 50%, and 75% were low, moderate, and high, respectively. Pooled results are presented as rate % (95% confidence interval (CI)).

## Results

### Literature search

**Fig. 1:**
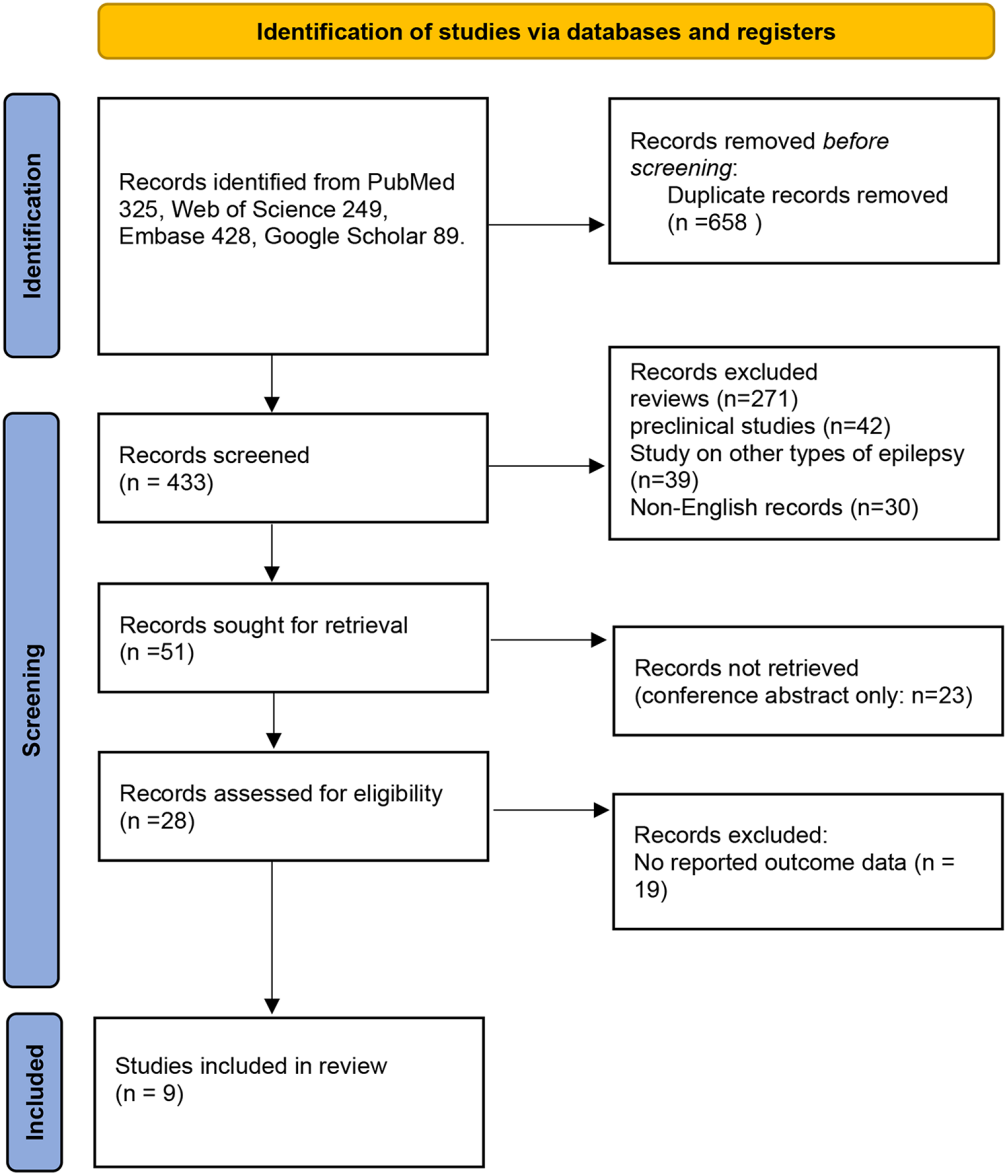
PRISMA flow chart

The literature search yielded 1091 references (PubMed: 325; Web of Science 249, Embase 428, Google Scholar 89). 658 duplicates were identified. Of the 433 reports screened, the full texts of 28 articles were reviewed for eligibility. Of these, 9 articles were excluded. Only nine full-text articles [17–19, 24–29] met the selection criteria (Fig. 1).

We included patients with a diagnosis of GGE in line with the ILAE criteria. The risk of bias was rated according to the NOS. Only one study included control groups and received a score of eight, six studies were assigned a score of seven and two studies received a score of six (Appendix 1). The risks of bias were low in seven studies. Two studies were found to be of moderate bias. Since all were observational studies, the available evidence is considered low per the GRADE working group criteria [23]. Details of the included papers are reported in Table 1.

**Table 1:** Summary of studies included in the systematic review

Study	Study type	GGE/Total sample size *N*.	Age (yrs) mean (SD)	Study duration (months) Mean (SD)	GGE Epilepsy type	GGE Responders *N*.	Responder rate of GGE %	GGE seizure-free *n*	AE %	Discontinued *N*. (%)
Devinsky, 1994	Prospective cohort	10/10	23.7 (5–37)	16.2 (5–31)	IGE (Absence± GTC)	8 4	80 50	1	60	1 (10)
Zupanc, 2010	Retrospective cohort	6/38	8	48	EMAtS 6	6	100	4	24	1 (2.6)
Shah, 2016	Retrospective cohort	62/103	10.7 (13.2)	35 (45)	IGE 53 Absence 9	25	42	-	17.5	8 (7.8)
Avanzini, 1996	Open label	25/379	22.8 (15)	5.9 (2.5)	GGE 25	15	60	3	-	-
Cilio, 2001	Open label	6/36	Median 9 (2.8–27)	36	GGE 6	2	33.3	0	63.8	0
Heyman, 2014	Retrospective cohort	2/50	5.5	13.2	EMAtS 1 CAE 1	1	50	0	44	20 (40)
Grosso, 2008	Retrospective cohort	10/53	2.7	25	EMAtS 8 EMA 2	8	80	5	30	0
Reed, 2024	Retrospective cohort	37/37	children	-	EMAtS 37	23	62	9	-	-
Kearney, 2009	retrospective cohort	8/13	36	> 36	GGE	5	62.5	0	53.8	1(7.7)

GGE: genetic generalized epilepsy; EMAtS: epilepsy with myoclonic-atonic seizures; EMA: epilepsy with myoclonic absences; CAE: childhood absence epilepsy; JME: juvenile myoclonic epilepsy; AE: adverse events

### Efficacy of FBM on GGE

Studies of the efficacy of FBM specifically on GGE are rare. Some earlier studies were interrupted or suspended due to the black box warning from the FDA in Aug 1994. FBM has been used as a second or third-line agent to treat patients with different types of DRE including GGEs in many centers but no detailed outcomes were reported. Our search resulted in nine studies including three open-label, six retrospective observational studies. Five studies included only pediatric patients. Four studies included both adult and pediatric patients. Two studies were specifically designed to study the effect of FBM on GGE. All the other studies included patients with all seizure types. The duration of study ranges from 3 months to 4 years. Certain patients were monitored for a duration of up to 20 years. All patients had DRE and had failed multiple ASMs at the commencement of FBM. All pediatric patients started with a dose of 10 mg/kg/day and titrated up to 45 mg/kg/day. In two studies, the dosage was up to 100 mg/kg/day [24, 25]. Adult patients were on 2400–3600 mg daily. In all the studies, FBM was used as an add-on therapy.

**Fig. 2:**
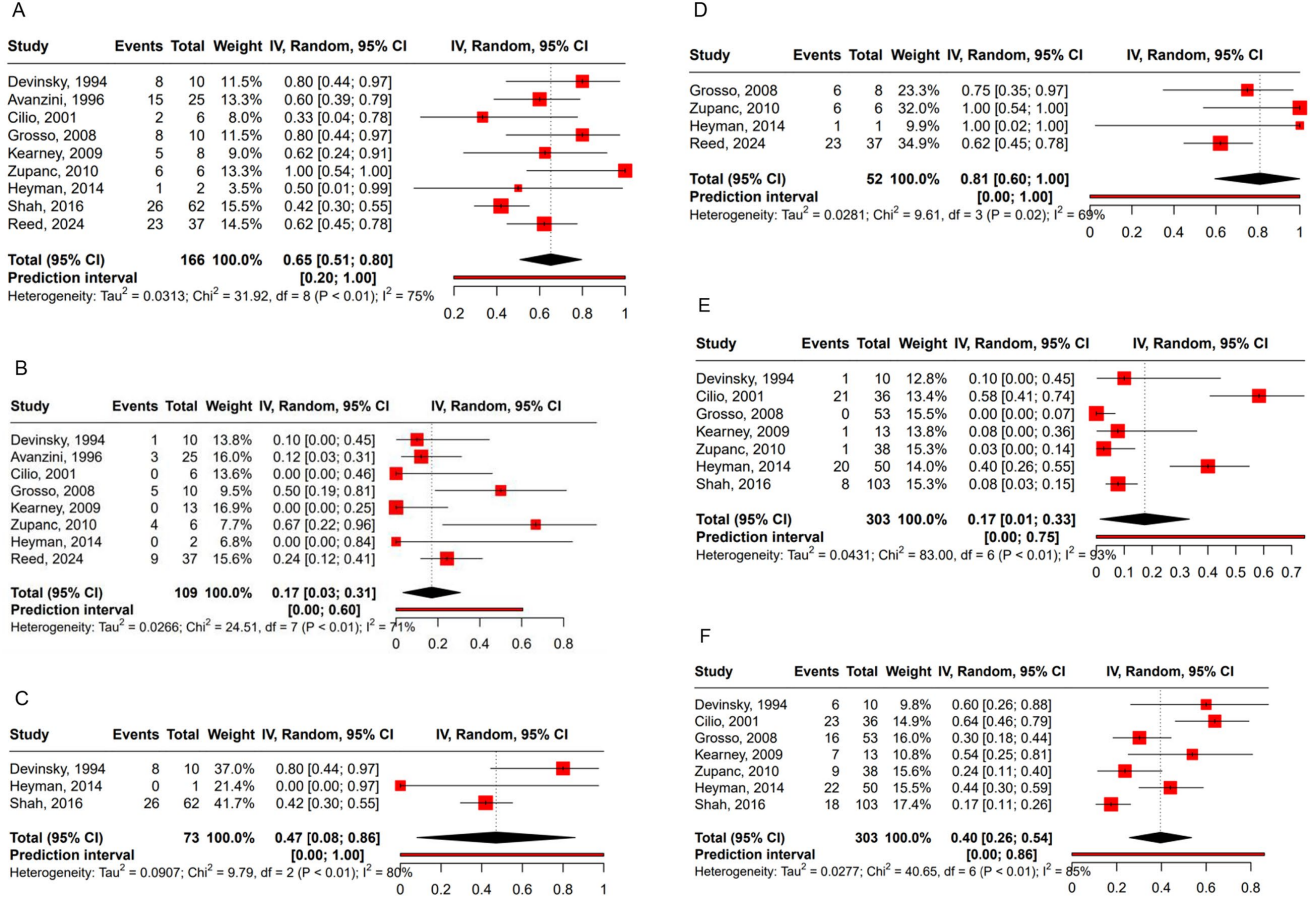
Outcome of patients treated with felbamate. (**A**) Responder rate; (**B**) Seizure freedom rate; (**C**) IGE responder rate; (**D**) EMAtS responder rate; (**E**) Dropout rate; (**F**) Advent events rate. High heterogeneity was found between studies. C.I., confidence interval

Outcome data of 166 patients with GGE were available for pooled analysis including 73 patients (44.0%) with IGE, 52 patients (31.3%) with EMAtS, two patients (1.2%) with EMA, and 44 patients (26.5%) with other GGEs with no documented specific epilepsy syndrome. The pooled responder rate to FBM was 65% (95% CI, 51–80) (Fig. 2A). 17% ( 95% CI, 3–31) achieved seizure freedom (Fig. 2B). We found significant intragroup heterogeneity in the responder rate (*I²=*75%, *P* < 0.01). Subgroup analysis showed the responder rate for IGE was 47% (95% CI, 8–86) and 81% (95% CI, 60–100) for EMAtS (Fig. 2C, D). The two cases of EMA were both responders. The significant heterogeneity among all studies is likely attributable to limited sample sizes and varying treatment protocols.

Retention rate can serve as a surrogate marker for the efficacy and tolerability of a drug. The retention rate of FBM was 83% (95% CI, 69–97) (Fig. 2E). There was high heterogeneity (*I²*=93%, *p* < 0.01) between studies, to which two studies mostly contributed [18, 25]. Both studies were open-label and commenced recruitment prior to the FDA warning. Both studies were interrupted, and many patients discontinued FBM due to fear of AA and hepatic failure, resulting in an elevated dropout rate.

### Adverse events (AEs) associated with FBM therapy

**Fig. 3:**
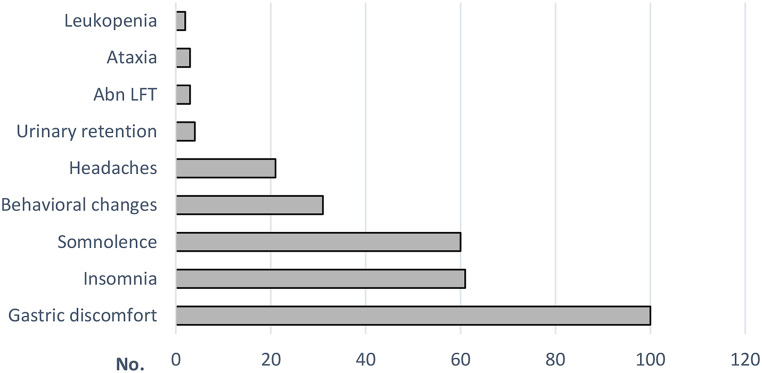
Reported adverse events. Gastric discomfort, including decreased appetite, anorexia, nausea, and mild weight loss, was the most common adverse event reported, followed by insomnia and somnolence

All the studies concluded that FBM was safe and well tolerated. No patients developed AA or hepatic failure. All the studies started after the FDA warning included intense lab monitoring protocol. Three (1.8%) developed elevated liver enzymes and two (1.2%) developed leukopenia which were reversible after FBM discontinuation [17, 25]. Four patients developed mild decreased hemoglobin but continued FBM at a lower dosage [17]. Most of the side effects were mild and did not lead to discontinuation of FBM. The Cilio group reported the highest AE rate at 63.9%. None of them discontinued FBM due to AEs [18]. On pooled analysis, 40% (95% CI:26–54) of patients experienced AEs (Fig. 2F). Gastric discomfort, including anorexia, decreased appetite, nausea, and weight loss, was the most common AE reported by 100 (14.7%) patients. Other AEs consistently reported by all studies were insomnia (8.9%), somnolence (8.8%), and behavioral changes (4.5%) (Fig. 3). AEs were manageable in most patients by reducing the dosage of other concomitant ASMs or FBM. All studies revealed the interaction between FBM and other ASMs resulted in elevated levels of Phenytoin, Valproic acid, and carbamazepine-epoxide. Heyman’s group discovered that a rapid titration schedule may result in neurotoxicity, while AEs could be mitigated by implementing a slightly slower titration schedule [25].

## Discussion

We conducted a thorough systematic assessment of three decades of data regarding the efficacy and safety of FBM in the treatment of drug-resistant GGE. Treatment response was achieved in about two-thirds (65%) of patients with seizure freedom achieved in 17%. There was not enough data for statistical analysis regarding how FBM affects different seizure types of GGE. FBM was quite beneficial for patients with EMAtS, with a pooled response rate of 81%. One study found an 80% response rate for absence seizures [28]. FBM is most likely effective for different seizure types of GGE. Most AEs were attributed to drug-drug interactions and were mitigated by decreasing the dosage of concurrent antiseizure medications. Despite the FDA’s black box warning, FBM’s overall retention rate remained at 83%, demonstrating its strong efficacy and good tolerability. There was significant heterogeneity between studies. This considerable heterogeneity is probably caused by small sample sizes, various types of epilepsy, and treatment approaches. All the studies started after the FDA warning included intense lab monitoring protocol. Rare cases of elevated liver function or mild leukopenia spotted early were reversible after discontinuation of FBM. None developed AA or hepatic failure. Close lab monitoring may be able to identify aberrant instances early on and prevent the development of AA or hepatic failure. There were not enough cases included to draw any conclusions. Larger-scale research is required to validate this conclusion. No random controlled trials (RCTs) were identified, and all the included studies were descriptive or observational. These results should be interpreted with caution.

FBM has a unique dual mechanism of action that blocks excitatory (NMDA) and potentiates inhibitory (GABA) receptors [30]. It has also been demonstrated to block voltage-gated sodium and calcium channels in a use-dependent manner [31, 32]. In addition, FBM was discovered to be a weak inhibitor of carbonic anhydrase and may suppress synaptosome adenosine reuptake [33]. This combination of mechanisms may account for its broad spectrum of anti-seizure activity [30]. FBM demonstrated little affinity for the GABA a-benzodiazepine receptor complex [34]. This may explain its absence of sedative and cognitive adverse effects [35]. FBM, in fact, exhibits stimulant-like effects that result in reduced appetite, weight loss, and sleeplessness [36]. This effect can be troublesome to some patients but can provide neurocognitive and metabolic benefits to others. Responders were reported to have improved attention, concentration, and alertness as well as social and motor skills [18]. This may result from decreased seizures or a direct drug effect. In our study, 8.8% of patients reported somnolence, potentially attributed to elevated levels of concomitant ASMs or FBM due to drug-drug interactions. Many studies did not record the concomitant ASMs and their levels when reporting AEs. The discrepancy in reporting criteria likely contributes to the high heterogeneity (I² =85%, *P* < 0.01) across all the studies.

Many patients with GGE are young and at a critical age for global development. Uncontrolled seizures can affect physical and neuropsychological development, causing mood disorders, repeated falls, serious trauma, status epilepticus, and sudden unexplained death in epilepsy (SUDEP). Treatment of refractory GGE can be challenging with limited treatment options. These patients are not good candidates for epilepsy surgery. Many ASMs traditionally used to treat focal seizures can make seizures in GGE worse. Carbamazepine (CBZ), oxcarbazepine (OXC), tiagabine (TGB), and vigabatrin (VGB) may worsen absence seizures and even cause absence status [37, 38]. Lamotrigine, lacosamide, gabapentin and pregabalin may trigger or worsen myoclonus [39–41]. Valproic acid (VPA) is effective for most patients but its use is limited due to the black box warning of hepatotoxicity, teratogenicity, and pancreatitis [42–44]. Topiramate and zonisamide are poorly tolerated due to cognition side effects. Levetiracetam, brivaracetam and perampanel can cause significant behavioral changes [45]. There is a substantial demand for alternative ASMs for patients with GGE. Clinicians should consider FBM as a therapeutic option for these patients as better seizure control can improve quality of life and decrease mobility and mortality associated with epilepsy. Due to the rare idiosyncratic reaction from FBM and drug-drug interactions, close laboratory monitoring and a slower titration schedule are recommended to help minimize AEs and improve tolerability.

There are several limitations to our study design. Six included studies were retrospective studies which can cause selection bias, recall bias, and missing data. The included cases are too small to screen for rare and fatal adverse events. All patients were administered multiple ASMs, complicating the assessment of felbamate’s actual effectiveness in enhancing seizure control. None of the included studies were RCTs. The absence of randomization frequently results in confounding bias, potentially distorting the actual factors associated with the outcome. The strength of our study is the updated systematic review of currently available data regarding the treatment of GGE with FBM. While our investigation encompassed a limited patient cohort and does not permit definitive conclusions, it offers supplementary clinical insights into the utilization of FBM as a therapeutic alternative for individuals with drug-resistant GGE.

## Conclusion

FBM has been administered to patients with drug-resistant GGE across multiple centers, demonstrating favorable response rates and low toxicity. Although FBM is not classified as a first-line ASM, epileptologists should acknowledge its potential therapeutic benefits. Future large-scale RCTs will be essential to ascertain the efficacy and safety of FBM as a therapeutic option for GGE.

## Electronic supplementary material

Below is the link to the electronic supplementary material.

Supplementary Material 1

## Declarations

### Disclaimer

The views expressed in the article are those of the authors and do not reflect the official policy of the Department of Army/Navy/Air Force, Department of health agency or the US government.
