# Correction To: Felbamate as a therapeutic alternative to drug-resistant genetic generalized epilepsy: a systematic review and meta-analysis

**DOI:** 10.1007/s10072-025-08229-0

**Published:** 2025-05-10

**Authors:** Yitao Ma, Matthew Kaminski, Robert Crutcher

**Affiliations:** 1 Department of Neurology, Walter Reed National Military Medical Center, Bethesda, MD 20889 USA; 2 University of Maryland, College Park, MD 20742 USA; 3 The Division of Neurology at Nemours, Mayo Clinic, Jacksonville, FL 32207 USA

**Correction to Neurological Sciences (2025) 46:1565–1572**

10.1007/s10072-024-07942-6

The article Felbamate as a therapeutic alternative to drug-resistant genetic generalized epilepsy: a systematic review and meta-analysis, written by Yitao Ma, Matthew Kaminski and Robert Crutcher, was originally published Online First without Open Access. After publication in volume 46, issue 4, pages 1565–1572 the author decided to opt for Open Choice and to make the article an Open Access publication. Therefore, the copyright of the article has been changed to © The Authors, 2025 and the article is forthwith distributed under the terms of the Creative Commons Attribution (CC BY) license 4.0 International License, which permits use, sharing, adaptation, distribution and reproduction in any medium or format, as long as you give appropriate credit to the original author(s) and the source, provide a link to the Creative Commons licence, and indicate if changes were made. The images or other third party material in this article are included in the article’s Creative Commons licence, unless indicated otherwise in a credit line to the material. If material is not included in the article’s Creative Commons licence and your intended use is not permitted by statutory regulation or exceeds the permitted use, you will need to obtain permission directly from the copyright holder. To view a copy of this licence, visit http://creativecommons.org/licenses/by/4.0.

The original article has been corrected.

