# Supplementary material for: Felbamate as a therapeutic alternative to drug-resistant genetic generalized epilepsy: a systematic review and meta-analysis
Source: Neurol Sci. 2024 Dec 26;46(4):1565–72. doi: 10.1007/s10072-024-07942-6 (PMC12064447; doi:10.1007/s10072-024-07942-6)
Supplement: Supplementary file 1 — Supplementary Material 1 [file 10072_2024_7942_MOESM1_ESM.docx]

**Felbamate as a Therapeutic Alternative to Drug-Resistant Genetic Generalized Epilepsy:**

**A Systemic Review and Meta-Analysis**

# Yitao Ma^1^, Matthew Kaminski^2^, Robert Crutcher^3^

# 1. Department of Neurology, Walter Reed National Military Medical Center, Bethesda, MD 20889

# corresponding author, mayt21054@gmail.com

# 2. University of Maryland, College Park, MD 20742

# 3. Mayo Clinic, Jacksonville, FL 32207

Appendix 1: Assessment of methodological quality of included studies by the Newcastle-Ottawa Scale.

| Reference | Representativeness of the exposed cohort | Selection of the nonexposed cohort | Ascertainment of exposure | Demonstration that outcome of interest was not present at the start of the study | Comparability of the cohorts included | Assessment of outcome | Was follow-up long enough for outcomes to occur? | Adequacy of follow-up of cohorts | Total score  >7, low risk  <6 mod risk |
| --- | --- | --- | --- | --- | --- | --- | --- | --- | --- |
| Devinsky, 1994 | * | - | * | * | * | * | * | * | ******* |
| Zupanc, 2010 | * | - | * | * | * | * | * | * | ******* |
| Shah, 2016 | * | - | * | * | * | * | * | * | ******* |
| Avanzini, 1996 | ? | - | * | * | * | * | * | * | ****** |
| Cilio, 2001 | * | - | * | * | * | * | * | * | ******* |
| Heyman, 2014 | * | - | * | * | * | * | * | * | ******* |
| Grosso, 2008 | * | - | * | * | * | * | * | * | ******* |
| Reed, 2024 | * | * | * | * | * | * | * | * | ******** |
| Kearney, 2009 | ? | - | * | * | * | * | * | * | ****** |
